# Risk factors associated with post-transplant superior caval vein stenosis in paediatric heart transplantation

**DOI:** 10.1017/S1047951121000573

**Published:** 2021-02-24

**Authors:** Hanna J. Tadros, Joseph T. Whelihan, Dalia Lopez-Colon, James C. Fudge, Himesh V. Vyas, Fredrick J. Fricker, Biagio A. Pietra, Mark S. Bleiweis, Dipankar Gupta

**Affiliations:** Congenital Heart Center, Department of Pediatrics, University of Florida, Gainesville, FL, USA

**Keywords:** Paediatric heart transplantation, superior caval vein stenosis, superior caval vein stenosis intervention

## Abstract

Superior caval vein stenosis is a known complication following paediatric heart transplantation. Herein, we sought to assess the incidence of superior caval vein stenosis and need for intervention in a single centre paediatric heart transplantation programme. A retrospective review was performed to identify variables associated with superior caval vein stenosis and need for intervention. Patients were identified based on angiographic and echocardiographic signs of superior caval vein stenosis. Of 204 paediatric heart transplantation recipients, 49 (24.0%) had evidence of superior caval vein stenosis with no need for catheter intervention and 12 (5.9%) had superior caval vein stenosis requiring catheter intervention. Overall, patients with superior caval vein stenosis with and without intervention had more cavopulmonary anastomosis (41.7%; 20.4%), pre-transplant superior caval vein procedures (41.7%; 28.6%), and bicaval approach (100.0%; 98.0%), compared to the group with no stenosis (11.9% and p = 0.015, 12.6% and p = 0.004, 73.4% and p < 0.001, respectively). Smaller recipients and donors were more likely to need intervention. Intervention was also seen more frequently in recipients who were younger at diagnosis (4.7 years) compared to non-intervention (13.3 years; p = 0.040). Re-intervention was required in 16.7% patients (n = 2) and was not associated with any complications.

Paediatric orthotopic heart transplantation is the gold-standard treatment for end-stage heart failure and irreparable congenital heart disease (CHD). Nonetheless, paediatric heart transplantation has several potential complications. One significant complication is superior caval vein stenosis leading to superior caval vein syndrome, a condition that may necessitate intervention under certain circumstances.^1^

Current data suggests pre- and intra-transplant variables may have effects on the development of superior caval vein stenosis.^2^ In regards to surgical technique, the introduction of the bicaval approach provided a promising alternative to biatrial techniques, with benefits seen in post-operative tricuspid valve regurgitation, early mortality and atrial pressures, and lower need for pacemaker placement.^3–5^ However, this technique has been hypothesised to increase the incidence of superior caval vein stenosis at the site of anastomosis. Additionally, previous superior caval vein surgical intervention, pre-transplant cavopulmonary anastomosis, donor and recipient caval size mismatch, and recipient weight and age have been recognised as possible risk factors.^2,6–8^

Previous studies have researched the relationship between paediatric heart transplantation and superior caval vein stenosis, but the body of data is limited by small sample sizes. Therefore, there is benefit from additional analyses to better delineate risk factors. Further, most studies have only analysed groups requiring intervention. We set out to identify patients whom had echocardiographic and/or angiographic evidence of superior caval vein stenosis and then went on to require intervention. We then identified pre- and intra-transplant variables associated with superior caval vein stenosis to identify potential risk factors. Finally, we analysed the efficacy and safety of interventions for the development of post-transplant superior caval vein stenosis.

## Methods

### Study cohort and outcomes

In this Institutional Review Board-approved study, we conducted a retrospective review of all paediatric heart transplantation recipients between 1988 and 2018 at the Congenital Heart Centre, University of Florida. Inclusion criteria included: (1) patients undergoing paediatric heart transplantation < 18 years; and (2) at least 1 year of follow up data available at our centre. Exclusion criteria were: (1) patients lost to follow up or transferred to another centre prior to 1 year; and (2) recipients with incomplete UNOS donor data in medical records and/or UNOS database. Study was approved with a full waiver of informed consent.

### Identification of superior vena cava stenosis

To identify those with superior caval vein stenosis, echocardiographic evidence of superior caval vein-right atrial gradient and turbulent flow by color Doppler was assessed in all recipients from echocardiogram reports. Additionally, post-transplant catheterisation reports were used to identify gradients invasively. Superior caval vein stenosis was defined as echocardiographic evidence of turbulent flow at the superior caval vein anastomosis site with a mean gradient > 1mmHg.^9^ Patients with evidence of gradients were placed into the superior caval vein stenosis group. To analyse risk factors for progression of superior caval vein stenosis needing intervention, patients were then assessed for eventual need for intervention using balloon angioplasty and/or stent placement. As such, three different groups were created: (a) No superior caval vein stenosis; (b) superior caval vein stenosis without intervention; and (c) superior caval vein stenosis with intervention.

### Echocardiographic and catheterisation follow up

Following paediatric heart transplantation patients receive an echo on the first day post transplant, and then done daily for 5 days followed by twice weekly until discharge. Subsequently, an echo is done at every outpatient visit. Patients are followed weekly for 4 weeks post-discharge and then biweekly for one more month. Cardiac catheterisation for older children is done at 2–3 weeks, 3 months, 6 months, and 1 year. For infants and smaller children (<4 years) catheterisation is done as needed (concern for rejection, possible need for intervention, etc.)

### Variables examined

The primary outcome was to assess donor and recipient risk factors associated with superior caval vein stenosis necessitating intervention. Risk factors assessed included demographics, pre-transplant superior caval vein surgical procedures, cavo-pulmonary anastomosis, and biatrial or bicaval transplant approach. Other variables at time of transplant like recipient and donor age, weight, height, and body mass index, were assessed. Those that eventually required intervention for superior caval vein stenosis were assessed separately and clinical courses were summarised.

### Statistics

Continuous variables were displayed as medians with interquartile ranges and categorical variables as counts with percentages and 95% confidence intervals of proportions. Kruskal–Wallis test was applied to compare the differences between three groups or more with data with non-normal distribution. Categorical variables were compared with Chi Square and Fisher exact test. Nonparametric data was compared using Mann–Whitney tests and normally distributed data with Student’s t tests. All statistics were performed using SPSS Version 25 (SAS Institute, Cary, NC, USA) and OpenEpi (Version 3.01).^10^

## Results

### Study cohort

Following exclusion criteria, 204 eligible pediatric heart transplant recipients were identified, of which 143 (70.1%) did not have any evidence of superior caval vein stenosis and 61 (29.9%) developed echocardiographic or angiographic evidence of superior caval vein stenosis. Of these, 49 (80.4%) had spontaneous resolution in superior caval vein stenosis without any intervention, and 12 (19.6%) required intervention for superior caval vein stenosis. Based on our experience, the incidence of superior caval vein stenosis requiring intervention in our cohort was 5.8% similar to previously reported data. Demographics, clinical features, and recipient and donor variables are summarised in Table 1.

### Pre-transplant interventions and bicaval transplant approach is associated with increased incidence of post-transplant superior caval vein stenosis

Our findings exhibited significantly higher prevalence of cavopulmonary anastomosis in patients who developed superior caval vein stenosis with and without intervention (41.7% (19.3, 68.1); n = 5 and 20.4% (11.48, 33.64); n = 10, respectively) compared to those with no superior caval vein stenosis (11.9% (7.6, 18.2); n = 17, p = 0.017). This association remained when patients were stratified by Glenn procedure, but not based on Fontan procedure (p > 0.05). Those with superior caval vein stenosis with and without intervention had a higher prevalence of Glenn procedure (41.7% (19.3, 68.1); n = 5 and 18.4% (10.0, 31.4); n = 9, respectively) compared to the non-stenosis group (11.2% (7.0, 17.4); n = 16, p = 0.014). Further, a higher prevalence of pre-transplant surgical superior caval vein procedures was found in patients who developed superior caval vein stenosis with and without intervention (41.7% (19.3, 68.1); n = 5 and 28.6% (17.9, 42.4); n = 14, respectively) compared to those with no superior caval vein stenosis (12.6% (8.1, 19.0); n = 18, p = 0.004). In regards to transplant surgical approach, a bicaval approach was more commonly taken in those whom developed superior caval vein stenosis with and without intervention (100.0%; n = 12 and 98.0% (89.3, 99.6); n = 48, respectively), compared to those with no stenosis (73.4% (65.6, 80.0); n = 105, p < 0.001). Groups with superior caval vein stenosis had higher prevalence of pre-transplant superior caval vein surgical intervention, cavopulmonary anastomosis, and bicaval approach.

### Recipient and donor variables may affect future development of superior caval vein stenosis

Our findings exhibited that the cohort with superior caval vein stenosis who underwent intervention had significantly lower recipient weight and height compared to the groups without superior caval vein stenosis and with superior caval vein stenosis not requiring intervention (p = 0.038 and p = 0.031, respectively; Table 2). Similar findings were seen when assess donor variables, with lower donor weight, height, body mass index, and age were seen in our group with superior caval vein stenosis (p = 0.015, p = 0.025, p = 0.029, and p = 0.033, respectively; Table 2). Donors to recipient weight ratios were found to be similar in all three groups. Findings suggest that lower recipient weight and height as well as younger donors and lower donor weight, height, and body mass index, increase the likelihood of development of significant superior caval vein stenosis.

### Recipient and donor variable differences in superior caval vein stenosis groups

To better identify variables associated with necessity for intervention, we preformed group-to-group comparison of intervention and non-intervention groups. Those who required intervention had lower recipient weight and height (p = 0.030 and p = 0.040, respectively) and lower donor weights and height (p = 0.022 and 0.039, respectively). Recipient body mass index was also lower in the intervention group compared to the non-intervention group (15.0 kg/m2 (14.1, 17.4) versus 19.5 kg/m2 (15.7, 23.5); p = 0.034). Further, donor body mass index was lower in the intervention group with statistical differences trending towards significance (17.8 kg/m2 (16.0, 20.3) versus 20.7 kg/m2 (18.5, 23.0); p = 0.068). Recipient age at time of paediatric heart transplantation was younger in the intervention group, although non-significant (3.5 years (0.33, 25.6) versus 12.1 years (0.19, 18.7); p = 0.121). Donor age at transplant was younger in the intervention group (p = 0.032) and those that eventually required intervention had a younger age at diagnosis (4.7 years (1.8, 10.0)) compared to the non-intervention group (13.3 years (5.8, 16.9); p = 0.040). Findings suggest that younger donor, younger age at diagnosis, and lower recipient and donor weight, height, and body mass index make intervention more likely.

### Intervention for superior caval vein stenosis is safe and effective

**Figure 1.**
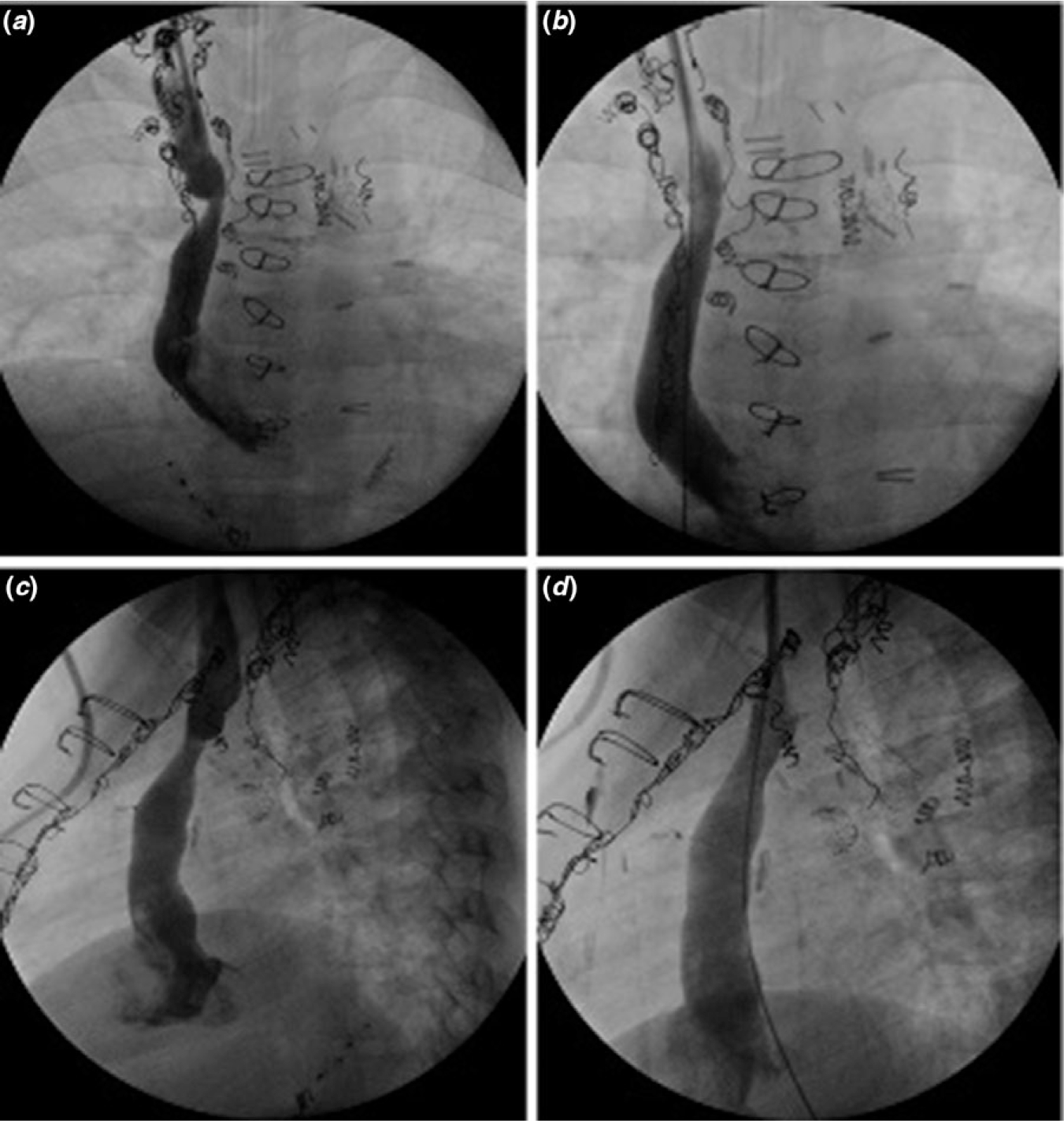
*(**a**)* Pre intervention anteroposterior angiogram demonstrating superior caval vein stenosis *(**b**)* Post intervention anteroposterior angiogram demonstrating angiographic resolution of superior caval vein stenosis *(**c**)* Pre intervention lateral angiogram demonstrating superior caval vein stenosis *(**d**)* Post intervention lateral angiogram demonstrating angiographic resolution of superior caval vein stenosis.

Of the 12 patients that required intervention, 75.0% (n = 9) received balloon angioplasty as first intervention and 8.3% (n = 1) required future stent placement. In total 33.3% (n = 4) patients required a stent angioplasty. No complications occurred during these procedures, except for one patient that had a small contained aneurysm (8.3%; n = 1) and median time from orthotopic heart transplantation until intervention was 0.24 years (0.15, 0.41). Further, in total, 16.7% (n = 2) required re-intervention and median days until re-intervention was 94.5 (50.8, 138.0). Following re-intervention, there were no indications that the superior caval vein stenosis had an adverse effect on patient disease course. One patient (8.3%; Patient 12) did not have a successful stent placement due to complete superior caval vein obstruction and only one patient (8.3%; Patient 12) had clinical symptoms manifesting as superior caval vein syndrome. Patients requiring intervention and their characteristics are detailed in Table 3. Figure 1 is an example of superior caval vein stenosis pre- and post-intervention.

## Discussion

Our study is a large single centre experience evaluating effect of donor and recipient factors in development of superior caval vein stenosis after paediatric heart transplantation. The findings identified that pre-transplant superior caval vein surgery, cavopulmonary anastomosis, bicaval surgical approach, and smaller recipients increase this risk. Additionally, we found that donor weight, age, and body mass index also influence development of superior caval vein stenosis. Those diagnosed at younger ages with evidence of superior caval vein stenosis were more likely to need intervention. Additionally, we exhibited that the use of balloon angioplasty and stent placement are safe interventions and require minimal re-intervention.

Superior caval vein stenosis requiring intervention is not very commonly seen following paediatric heart transplantation. In our study, we found a significantly large patient population with echocardiographic and angiographic evidence suggestive of superior caval vein stenosis in 29.9% of patients. Nonetheless, this gradient largely did not progress and they remained asymptomatic. Although data on gradient resolution is lacking in the literature, we hypothesise that those with echocardiographic and/or angiographic signs of superior caval vein stenosis without need for intervention resolved over time with vessel growth and scar maturation. On the other hand, 5.9% of our patient population developed superior caval vein stenosis that required intervention with either balloon angioplasty or stent placement. This is similar to other pediatric studies which show a prevalence of 3.1%–5.1%.^2,7,9^ Interestingly, all patients requiring intervention had a bicaval approach and around 40% had pre-transplant cavopulmonary anastomosis and/or superior caval vein surgical intervention, echoing previously exhibited associations.^2,9^

Further, it has also been suggested that smaller recipients may be at increased risk of post-transplant superior caval vein stenosis.^7^ Our group requiring interventions was significantly smaller than both other groups in height and weight. Further, we were able to also exhibit that donor weight and height was also smaller in this group. Although previous studies suggest that younger age of recipient may be associated with superior caval vein stenosis, we found that younger donor age in particular was found in the intervention group. In the absence of significance differences in donor: recipient weight ratios, this may suggest that just smaller donor and recipient caval size may have more significant effect on development of superior caval vein stenosis, rather than caval mismatch contrary to previous studies.^8,11^

Within our intervention group, stent and balloon interventions were a safe treatment for superior caval vein stenosis. Intervention efficacy and safety has been described previously in the literature as well. Small studies showed that intervention was effective and safe.^12,13^ Larger studies exhibited that complications occurred in 0%–19% of patients and re-intervention was necessary in 22%–33%.^7,14^ In our study, none of our patients had any acute complications, except for a single episode of a contained small aneurysm and one patient where stent placement failed. However, 16.7% required re-intervention, and 66.7% required only balloon angioplasty without need for stenting.

Our study is limited by its retrospective nature and the small sample size of the intervention group, although this represents a large, single centre, pediatric transplant cohort with expected rates of significant superior caval vein stenosis requiring intervention. Future directions include multi-centre studies to establish further factors associated with heightened risk of superior caval vein stenosis and to better understand if caval mismatch may truly be a risk factor for superior caval vein stenosis.

In conclusion, pre-transplant cavo-pulmonary anastomosis, bicaval approach, and history of superior caval vein surgery increase risk of post-transplant superior caval vein stenosis. Additionally, smaller recipient and donor weight, height, and body mass index, as well as younger donor age all suggesting smaller caval size is exhibited in patients requiring intervention. Overall, most patients with echocardiographic evidence of superior vena caval gradient demonstrate spontaneous resolution. Intervention, if necessary, for superior vena caval stenosis is safe and effective, though may need re-intervention in some patients.

## Figures and Tables

**Table 1.** Demographics and clinical features of recipients

Demographics/Clinical features	n (%)	Recipient variables	n (%) or Median (IQR)
Gender		Weight at time of paediatric heart transplantation	25.1 kg (7.53, 50.0)
Male	114 (55.9%)	Height at time of paediatric heart transplantation	117.0 cm (69.6,153.0)
Female	90 (44.1%)	Body mass index at time of paediatric heart transplantation	18.0 kg/m^2^ (15.2, 23.1)
Race		Age at time of paediatric heart transplantation	9.18 years (0.96, 14.88)
Non Hispanic white	106 (52.0%)	Donor variables	
Hispanic white	31 (15.2%)	Weight at time of paediatric heart transplantation	40.0 kg (10.9, 63.3)
African American	59 (28.9%)	Height at time of paediatric heart transplantation	142 cm (81.3, 168.0)
Other	8 (3.9%)	Body mass index at time of paediatric heart transplantation	18.9 kg/m^2^ (16.0, 21.9)
Cavopulmonary anastomosis	32 (15.6%)	Age at time of paediatric heart transplantation	10.0 years (1.00, 17.0)
Previous superior caval vein surgery	37 (18.1%)	Surgical approach	
Superior caval vein stenosis (non-intervened)	49 (24.0%)	Bicaval	165 (80.8%)
Superior caval vein stenosis (intervened)	12 (5.9%)	Biatrial	39 (19.2%)

kg, kilograms; m, meter.

**Table 2.** Group comparisons of recipient and donor variables

	No superior caval vein stenosis	Superior caval vein stenosis (no intervention)	Superior caval vein stenosis (intervention)	p-value
Recipient variables				
Weight at time of paediatric heart transplantation	23.0 kg (3.3, 156.0)	30.0 kg (3.0, 109.0)	11.0 kg (3.5, 92.5)	0.038*
Height at time of paediatric heart transplantation	115 cm (48.0, 186.0)	135.0 cm (54.0, 180.0)	87.9 cm (51.0, 174.0)	0.031*
Body mass index at time of paediatric heart transplantation	18.1 kg/m^2^ (10.2, 52.2)	19.5 kg/m^2^ (10.3, 45.5)	15.0 kg/m^2^ (12.3, 30.4)	0.096
Age at time of paediatric heart transplantation	8.0 years (0.1, 19.0)	12.1 years (0.19, 18.7)	3.5 years (0.33, 25.6)	0.136
Donor variables				
Weight at time of paediatric heart transplantation	32.0 kg (10.0, 17.0)	56.0 kg (29.5, 70.0)	15.9 kg (10.5, 42.5)	0.015*
Height at time of paediatric heart transplantation	135.0 cm (77.8, 168.0)	162.0 cm (127.0, 172.0)	99.0 cm (74.3, 141.0)	0.025*
Body mass index at time of paediatric heart transplantation	18.5 kg/m^2^ (15.8, 21.7)	20.7 kg/m^2^ (18.5, 23.0)	17.8 kg/m^2^ (16.0, 20.3)	0.029*
Age at time of paediatric heart transplantation	9.0 years (1.25, 17.0)	15.0 years (8.0, 17.0)	3.0 years (1.8, 11.0)	0.033*
Donor: recipient weight ratio	1.2 (1.0, 1.7)	1.3 (1.0, 1.7)	1.4 (1.0, 1.7)	0.433

kg, kilograms; m, meter.

* p < 0.05.

**Table 3.** Intervention group characteristics

	Sex	Pre-transplant diagnosis	Surgical approach	Prior CPA	Recipient weight (kg)	Donor weight (kg)	Age at transplant (years)	Age at diagnosis (years)	Intervention	Pre-intervention findings	Post-intervention Success	Re-intervention
1	F	Hypoplastic Left Heart Syndrome s/p Fontan, plastic bronchitis, end-stage heart failure	Bicaval	y	29.0	50.0	10.0	10.0	Balloon (12 mm × 4 cm Mustang)	Superior caval vein Description: 5.1 mm versus 11.4 mm. Gradient: was noted to be present, but with no number	y	None
2	M	Transposition of the great arteries, criss-cross atrioventricular valves, superior/inferior ventricles, hypoplastic pulmonary arteries	Bicaval	n	8.4	12.4	1.3	1.6	Balloon (10 mm × 2 cm Mustang)	Superior caval vein Description: 4.7 mm versus 9.9 mm. Gradient: 5mmHg	y	None
3	F	Complete atrioventricular septal defect, interrupted inferior vena cava, left atrial isomerism, severe left ventricular outflow tract obstruction, complete atrioventricular block	Bicaval	n	3.5	3.5	0.5	0.5	Balloon (9 mm × 2 cm Sterling)	Superior caval vein Description: near total occlusion, diffuse, long segment stenosis, decompressing collaterals, retrograde flow into azygous vein. Gradient: 16mmHg	y	Stent (10 mm x 17 mm Bard Valeo)
4	M	Double-inlet left ventricle, transposition of the great arteries, s/p pulmonary artery banding, end-stage heart failure	Bicaval	n	10.6	19.5	4.4	4.5	Balloon (12 mm Atlas Gold)	Superior caval vein Dimensions: 7.1 mm versus 10.4 mm. Gradient: 6mmHg	y	None
5	M	Double outlet right ventricle, pulmonary atresia, heterotaxy syndrome, right atrial isomerism, unbalanced canal defect	Bicaval	n	3.7	6.2	0.3	0.7	Stent (7 × 18 mm Vispro)	Superior caval vein Dimensions: 5, 3 mm versus 24.1 mm. Gradient: 5mmHg	y	Balloon (8 mm x 2 cm Dorado)
6	M	Left ventricle non-compaction cardiomyopathy, end-stage heart failure	Bicaval	n	92.5	69.0	18.6	18.6	Balloon (16 mm × 4 cm Atlas Gold)	Superior caval vein Dimensions: 6.6 mm versus 9.8 mm. Gradient: 7mmHg	y	None
7	F	Hypoplastic left heart syndrome with double outlet right ventricle, s/p Fontan, end-stage heart failure.	Bicaval	y	20.0	27.2	9.8	10.0	Balloon (Atlas 16mm)	Superior caval vein Dimensions: 5.5 mm versus 6.5 mm. Gradient: 5mmHg	Improvement in dimensions, but gradient remains	None
8	F	Unbalanced atrioventricular septal defect, severe atrioventricular valve regurgitation, s/p Glenn, end-stage heart failure	Bicaval	y	11.4	11.8	2.7	2.8	Balloon (10 mm × 2 cm Sterling)	Superior caval vein Dimensions: 4.6mm versus 12.1 mm. Gradient: 5mmHg	y	None
9	M	Hypoplastic left heart syndrome, s/p Fontan, end-stage heart failure	Bicaval	y	37.3	66.8	16.6	16.6	Stent (36 mm Intrastent Max LD)	Superior caval vein Dimensions: 4.6 mm versus 12.1 mm Gradient: 8mmHg	y	None
10	M	Dilated cardiomyopathy, end-stage heart failure	Bicaval	n	9.9	12.2	1.8	1.8	Balloon (12 mm × 4 cm)	Superior caval vein Dimensions: 7.3 mm versus 10.1 mm. Gradient: 7mmHg	y	None
11	M	Dilated cardiomyopathy, end-stage heart failure	Bicaval	n	22.0	40.0	9.3	9.4	Balloon (14 mm × 4 cm)	Superior caval vein Dimensions: NA. Gradient: 4mmHg	y	None
12	M	Hypoplastic left heart syndrome, s/p Glenn, end-stage heart failure	Bicaval	y	6.0	6.4	0.9	4.9	Failed stent placement	Superior caval vein Dimensions: stenosis site 2.6 mm. Gradient: 6	Failed	None

F, female; kg, kilogram; M, male; s/p, status post.
